# Using Geochemistry, Stable Isotopes and Statistical Tools to Estimate the Sources and Transformation of Nitrate in Groundwater in Jinan Spring Catchment, China

**DOI:** 10.3390/toxics13050393

**Published:** 2025-05-14

**Authors:** Kairan Wang, Mingyuan Fan, Zhen Wu, Xin Zhang, Hongbo Wang, Xuequn Chen, Mingsen Wang

**Affiliations:** 1 Water Resources Research Institute of Shandong Province, Jinan 250014, China; wkrshmily@aliyun.com (K.W.); fantina715@126.com (M.F.); skywuzhen@shandong.cn (Z.W.); skyzhangxin@shandong.cn (X.Z.); waterwms@163.com (M.W.); 2 Shandong Provincial Key Laboratory of Water Resources and Environment, Jinan 250014, China; 3 School of Municipal and Environmental Engineering, Shandong Jianzhu University, Jinan 250101, China

**Keywords:** karst groundwater, hydrochemical characteristics, stable isotopes, nitrate, spatial distribution

## Abstract

Nitrate (NO_3_^−^) pollution resulting from anthropogenic activities represents one of the most prevalent environmental issues in karst spring catchments of northern China. In June 2021, a comprehensive study was conducted in the Jinan Spring Catchment (JSC), where 30 groundwater and surface water samples were collected. The sources and spatial distribution of nitrate pollution were systematically investigated through hydrochemical analysis combined with dual-isotope tracing techniques (δ^15^N_NO3_ and δ^18^O_NO3_). Analytical results revealed that the predominant anion and cation sequences were HCO_3_^−^ > SO_4_^2−^ > Cl^−^ > NO_3_^−^ and Ca^2+^ > Na^+^ > Mg^2+^ > K^+^, respectively, with HCO_3_·SO_4_-Ca identified as the primary hydrochemical type. Notably, the average NO_3_^−^ concentration in groundwater (46.62 mg/L) significantly exceeded that in surface water (4.96 mg/L). Among the water samples, 11 locations exhibited substantial nitrate pollution, demonstrating an exceedance rate of 42%. Particularly, the NO_3_^−^-N concentrations in both the upstream recharge area and downstream drainage area were markedly higher than those in the runoff area. The spatial distribution of NO_3_^−^ concentrations was primarily influenced by mixing processes, with no significant evidence of denitrification observed. The isotopic compositions ranged from −1.42‰ to 12.79‰ for δ^15^N_NO3_ and 0.50‰ to 15.63‰ for δ^18^O_NO3_. Bayesian isotope mixing model (MixSIAR) analysis indicated that domestic sewage and manure constituted the principal nitrate sources, contributing 37.1% and 56.9% to groundwater and surface water, respectively. Secondary sources included soil organic nitrogen, rainfall and fertilizer NH_4_^+^, and chemical fertilizers, while atmospheric deposition showed the lowest contribution rate. Additionally, potential mixing of soil organic nitrogen with chemical fertilizer was identified.

## 1. Introduction

Karst groundwater serves as a crucial and irreplaceable resource that supports industrial and agricultural production, as well as social development in Jinan, which is widely recognized as the Spring City [1]. Since the 1980s, the exploitation of karst groundwater in Jinan has progressively intensified in parallel with the accelerating urbanization process. Industrial and agricultural activities, tourism development, and domestic sewage discharge have collectively contributed to the contamination of karst groundwater, resulting in a gradual deterioration of water quality over time [2]. Particularly in the recharge and runoff areas of the spring domain, the convergence of point and non-point-source pollution in suburban and rural regions has rendered the issue of nitrate pollution increasingly prominent [3]. NO_3_^−^ represents a prevalent environmental contaminant that not only occurs naturally but is significantly influenced by anthropogenic activities [4,5,6,7]. Excessive NO_3_^−^ concentrations in drinking water can induce methemoglobinemia in children and elevate the risk of gastric and esophageal cancers in adults, thereby posing substantial threats to human health [8,9,10]. Concurrently, the karst region is characterized by a distinctive “surface-underground” dual structure, and the unique nature of its aquifer system determines that the NO_3_^−^ pollution scenario is particularly complex [11,12].

In recent years, numerous scholars have extensively investigated nitrate pollution in karst water systems, with particular emphasis on the subtropical karst regions of southwest China [13,14,15,16]. However, research on semi-humid and semi-arid karst regions in northern China remains relatively limited [17]. Previous studies in the Jinan spring area have predominantly focused on hydrogeological conditions, karst aquifer structures, water cycle characteristics, and the water environment [18,19,20]. In particular, concerning the hydrochemical characteristics and evolutionary processes of the karst groundwater environment, several researchers have conducted comprehensive analyses of major elements, trace elements, and stable environmental isotopes within the JSC karst groundwater system. These investigations have focused on critical aspects, including groundwater recharge sources and hydrogeochemical characteristics [21,22]. Furthermore, other studies have examined the patterns of water quality changes and their influencing factors in the JSC karst groundwater [23,24]. Generally, NO_3_^−^ in karst water has often derived from multiple pollution sources, which makes it challenging to quantitatively identify sources using traditional hydrogeochemical approaches, including the relationship between Cl^−^ and NO_3_^−^/Cl^−^ [25,26,27]. Nevertheless, research utilizing hydrochemical and NO_3_^−^ nitrogen–oxygen dual-isotope analysis to investigate the characteristics and sources of NO_3_^−^ pollution in karst groundwater systems at the spring basin scale remains relatively scarce [28,29].

Therefore, this study systematically investigates the hydrochemical characteristics of karst water in the JSC through a hydrogeochemical approach, employing hydrochemical analysis and NO_3_^−^ nitrogen–oxygen dual-isotope tracing techniques. It examines the sources, spatial distribution, and influencing factors of NO_3_^−^ contamination, quantitatively evaluates the proportional contributions of various NO_3_^−^ sources, and elucidates the biogeochemical processes of NO_3_^−^. The research aims to establish a scientific foundation for the sustainable development and utilization of karst water resources, as well as the protection of the ecological environment in the JSC.

## 2. Materials and Methods

### 2.1. Study Area

Jinan is located in the warm temperate continental monsoon climate zone, featuring an average annual temperature of 14.3 °C and an average annual precipitation of 667.1 mm. Precipitation is predominantly concentrated between June and September, exhibiting a spatial decrease from the southeast to the northwest. The major rivers that traverse Jinan encompass the Yellow River, the Yufu River, the Beidasha River, and the Xiaoqing River. The Yellow River courses through the northern sector of Jinan City, whereas the Yufu River and the Beidasha River merge into the Yellow River from the southern mountainous regions. The Xiaoqing River traverses the urban area from west to east, gathering Jinan’s spring water before it flows eastward into the Bohai Sea [3].

**Figure 1:**
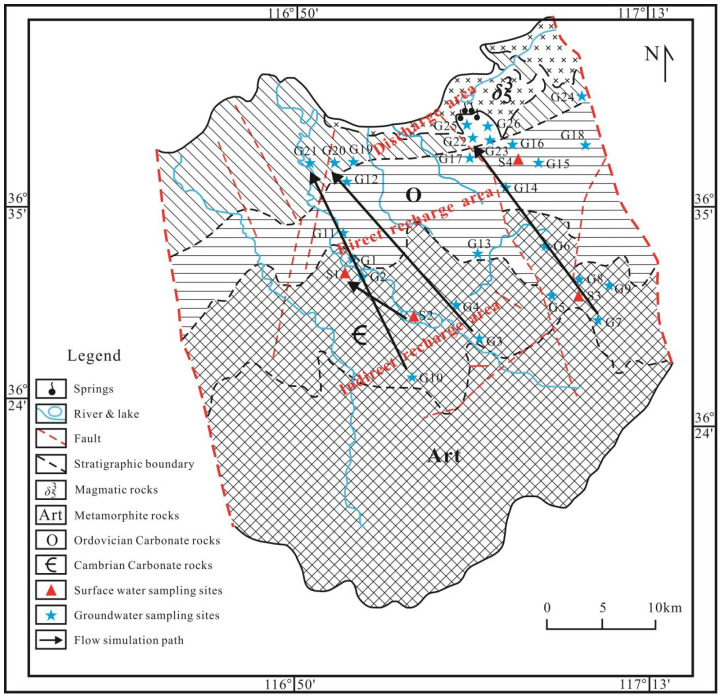
Hydrogeological sketch of the study area and location of water samples.

The JSC is characterized as a monocline geological structure exhibiting a gentle northward inclination, representing a prototypical karst development region in northern China, with a total coverage area of approximately 1500 km^2^ (Figure 1). The Paleozoic (Cambrian and Ordovician) carbonate formations are monocline structures, covering the metamorphic rock series and basically consistent with the topographic orientation. These strata are hidden beneath the Quaternary formations in the north [30]. In the northern, eastern, and western suburbs of the JSC, extensive gabbro bodies that intruded during the Yanshan period are widely distributed. West of the Yufu River, along the Yellow River area, the Ordovician carbonate formations are buried beneath the Carboniferous and Permian formations, extending in a northwest–southeast direction. The specific geological structural conditions primarily control the spatial distribution of aquifers and groundwater circulation in the JSC [31].

The karst aquifer system in the catchment, primarily composed of carbonate rock fractures, encompasses the Middle Cambrian Zhangxia Formation, the Upper Cambrian Fengshan Formation, and the Ordovician aquifer. The lithological composition includes limestone, argillaceous limestone, dolomitic limestone, calcareous dolomite, and dolomite, etc. The well-developed karst fractures and their excellent connectivity significantly enhance the recharge, runoff, and discharge processes of groundwater [32]. The JSC is systematically divided into three distinct zones from south to north: indirect recharge area, direct recharge area, and discharge area [23]. Upon receiving atmospheric precipitation, the karst water flows from south to north, where it encounters gabbro bodies, causing it to rise to the surface and form springs. The groundwater and surface water in the JSC exhibit a strong hydraulic connection, rendering the karst aquifer particularly vulnerable to mixed pollution from surface water sources [33,34].

Within the catchment area, human activities are predominantly centered on agricultural production, with the absence of large-scale polluting industrial facilities. Residential zones are dispersed in a scattered pattern, characterized by extensive poultry farming operations and the prevalent use of septic tank systems. In the southern recharge and runoff zones, agricultural land and forested areas constitute over 80% of the total regional coverage. The primary fertilization periods in this region occur during April and August, with chemical fertilizers, including compound fertilizers, urea, and ammonium bicarbonate, being predominantly utilized.

### 2.2. Sample Collection and Analysis

In June 2021, an extensive sampling campaign was implemented across the study area, resulting in the collection of 30 water samples, including 4 surface water and 26 groundwater specimens. The geographical distribution of sampling sites is depicted in Figure 1. All samples were acquired using a standardized liquid sampling apparatus [8], with surface water samples consistently collected at a depth of 0.5 m below the water–air interface. Groundwater samples were collected from deep aquifers through both civilian wells and professional monitoring wells, with all water samples characterized by well depths exceeding 150 m and water table depths greater than 60 m. For sealed wells, a 5 min pre-pumping protocol was implemented prior to sampling to ensure representative groundwater collection. In open wells, samples were collected at 0.5 m below the water surface using the liquid sampler. Field measurements of water quality parameters, including temperature (T), electrical conductivity (EC), total dissolved solids (TDS), dissolved oxygen (DO), and pH, were performed using a portable multi-parameter water quality analyzer (Hach HQ40D, Loveland, CO, USA) with respective measurement accuracies of 0.1 °C, 1 µS/cm, 0.01 mg/L, 0.01 mg/L, and 0.01 pH units. For laboratory analysis, water samples were collected in pre-cleaned 600 mL polyethylene bottles, ensuring the absence of air bubbles, for subsequent cation and anion concentration determinations. Cation analysis (K^+^, Na^+^, Ca^2+^, Mg^2+^) was conducted using inductively coupled plasma optical emission spectrometry (ICP-OES). Anion concentrations (Cl^−^, NO_3_^−^, SO_4_^2−^) were determined by ion chromatography with a detection limit of 0.01 mg/L [4]. Ammonium (NH_4_^+^) was quantified using the Nessler reagent colorimetric method, while nitrite (NO_2_^−^) was measured via the α-naphthylamine colorimetric method, both with 0.01 mg/L accuracy. Bicarbonate (HCO_3_^−^) and total hardness were determined using titration methods with a precision of 0.01 mg/L. All chemical analyses were conducted at the Experimental Testing Center of the Lunan Geological Engineering Exploration Institute, China.

For isotope analysis, water samples were collected in pre-cleaned 40 mL polyethylene bottles. On site, the samples were filtered through a 0.22 μm mixed cellulose membrane and subsequently refrigerated prior to transportation to the laboratory for nitrate δ^15^N and δ^18^O isotope analysis. During the analytical process, specific denitrifying bacteria were employed to convert nitrate nitrogen into N_2_O. The N and O isotopes of N_2_O were then measured using the ISOPRIME100-Tracegas trace gas isotope mass spectrometer (manufactured by Isoprime, UK). The instrument demonstrated a detection precision of 0.01‰, with standard deviations of <0.4‰ for δ^15^N measurements and <0.22‰ for δ^18^O measurements. The isotopic detection results were expressed in delta (δ) units, representing the deviations in per mil (‰) notation relative to international standards, as described by Equation (1):

*δ*_sample_ = (*R*_sample_/*R*_standard_ − 1) × 1000‰ (1)

where *R*_sample_ and *R*_standard_ represent the analytical isotopic ratios (^18^O/^16^O, or ^15^N/^14^N) for samples and standards, respectively. The ^18^O/^16^O value is reported relative to the Vienna Standard Mean Ocean Water (VSMOW), whereas the ^15^N/^14^N value is reported with respect to atmospheric N_2_ (AIR) [35]. The ^15^N and ^18^O isotopes were analyzed at the Agricultural Environmental Stable Isotope Laboratory, Chinese Academy of Agricultural Sciences, China.

### 2.3. Statistical Analysis

The Piper trilinear diagram was generated using Aq·QA v.1.1 software, while correlation coefficients of groundwater chemical components were calculated using MATLAB 9.3. Spatial visualization was achieved through GIS 10.3 for creating location maps and spatial distribution maps, and Origin 10.0 was employed for generating scatter plots.

This study identifies the sources of NO_3_^−^ by analyzing ^15^N and ^18^O isotope values and employs the stable isotope mixing model MixSIAR to quantify the contribution rates of various NO_3_^−^ sources. The MixSIAR model has been established as a reliable tool for NO_3_^−^ source apportionment [35,36]. The model framework, which accounts for *j* isotopes from *k* sources in *n* mixtures while considering isotopic fractionation effects, can be mathematically expressed by Equation (2) [37]:

(2) $$X_{ij}=\frac{\sum_{k=1}^{K}p_{k}q_{jk}\left(S_{jk}+C_{jk}\right)}{\sum_{k=1}^{K}p_{k}q_{jk}}+\varepsilon_{ij}$$

$$S_{jk}\sim N(\mu_{jk},\;\omega_{jk}^{2})$$

$$C_{jk}\sim N(\lambda_{jk},\;T_{jk}^{2})$$

$$\varepsilon_{ij}\sim N(0,\;\sigma_{j}^{2})$$

where *X_ij_* represents the value of the *j*-th isotope in the *i*-th sample (where *i* = 1, 2, 3, …, *N*; *j* = 1, 2, 3, …, *J*), *S_jk_* is the value of the *j*-th isotope in the *k*-th source (where *k* = 1, 2, 3, …, *K*), *μ*_jk_ is the mean value, $\omega_{jk}^{2}$ represents the variance of a normal distribution, *C_jk_* denotes the fractionation coefficient of the *j*-th isotope in the *k*-th source, and *λ_jk_* represents the mean value, while $T_{jk}^{2}$ indicates the variance of a normal distribution. *p_k_*, which is calculated by the model, represents the contribution rate of the *k*-th source, and *q_jk_* signifies the concentration of isotope *j* in the *k*-th source. *ε_ij_* represents the residual, accounting for the undetermined variables among the mixtures, with a mean of 0, and $\sigma_{j}^{2}$ denotes the variance of a normal distribution.

## 3. Results and Discussion

### 3.1. Hydrochemistry and Nitrate Pollution Characteristics

#### 3.1.1. Hydrochemistry Characteristics

The hydrochemical analysis of groundwater and surface water in the study area (Table 1) reveals that both water sources exhibit pH values predominantly exceeding 7, with a peak value of 8.72, demonstrating their weakly alkaline characteristics. Along the groundwater flow path, the TDS in karst groundwater display a decreasing trend, with concentrations ranging from 119.97 mg/L to 1226.05 mg/L. Elevated concentrations of Ca^2+^, Mg^2+^, SO_4_^2−^, and NO_3_^−^ were observed in water samples G4 and G6 and of NO_3_^−^ at G5, suggesting significant pollution of karst groundwater in these locations. Among the cations, Ca^2+^ and Mg^2+^ predominate, while HCO_3_^−^ and SO_4_^2−^ are the dominant anions, collectively constituting over 80% of the total cation and anion content in both groundwater and surface water.

**Figure 2:**
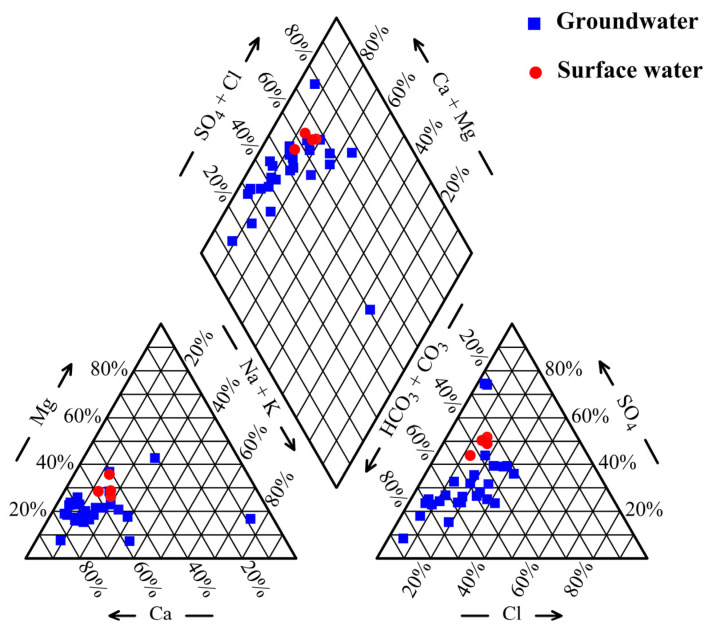
Piper diagram of groundwater and surface waters sampled in the study area.

The Piper trilinear diagram of the study area’s hydrochemistry (Figure 2) reveals that all water samples are located in the upper left quadrant of the diamond-shaped plot, where alkaline earth metals (Ca, Mg) predominate over alkali metals (K, Na). This indicates that the groundwater chemistry is primarily characterized by alkaline earth metals and weak acids. Based on statistical data analysis in conjunction with the Piper trilinear diagram, it is apparent that the dominant anions and cations in both groundwater and surface water of the study area are HCO_3_^−^ and Ca^2+^, respectively. According to the Shukarev classification system, the predominant hydrochemical type in the study area is HCO_3_·SO_4_-Ca, representing 60% of all samples and primarily distributed in the runoff and discharge zones. The HCO_3_-Ca type accounts for 17% of the total samples. Additionally, the study area exhibits a complex hydrochemical system, as evidenced by the presence of various water types, including HCO_3_-Ca·Mg, HCO_3_·SO_4_-Ca·Mg, HCO_3_·SO_4_·Cl-Ca·Na, SO_4_-Ca·Mg, and SO_4_·Cl-Ca.

#### 3.1.2. Nitrate Pollution Characteristics

The average concentration of NO_3_^−^ in the groundwater of the JSC (46.62 mg/L) significantly exceeds that in the surface water (4.96 mg/L). In comparison with groundwater quality standards, analysis of 26 groundwater samples revealed the following distribution: 12% of the samples exhibited NO_3_^−^-N concentrations ≤ 2.0 mg/L, 15% showed concentrations between 2.0–5.0 mg/L, and 65% demonstrated concentrations between 5.0–20 mg/L. Notably, 8% of the samples contained NO_3_^−^-N concentrations > 20 mg/L. When evaluated against the World Health Organization’s NO_3_^−^-N concentration guideline of 10 mg/L [38], 42% of the samples exceeded this threshold. In contrast, all four surface water samples maintained NO_3_^−^-N concentrations below 2 mg/L, substantially lower than the ≤10 mg/L limit established for centralized drinking water surface water sources in surface water environmental quality standards. These findings collectively suggest a potential risk of point-source pollution in the study area’s groundwater quality during the dry season.

**Figure 3:**
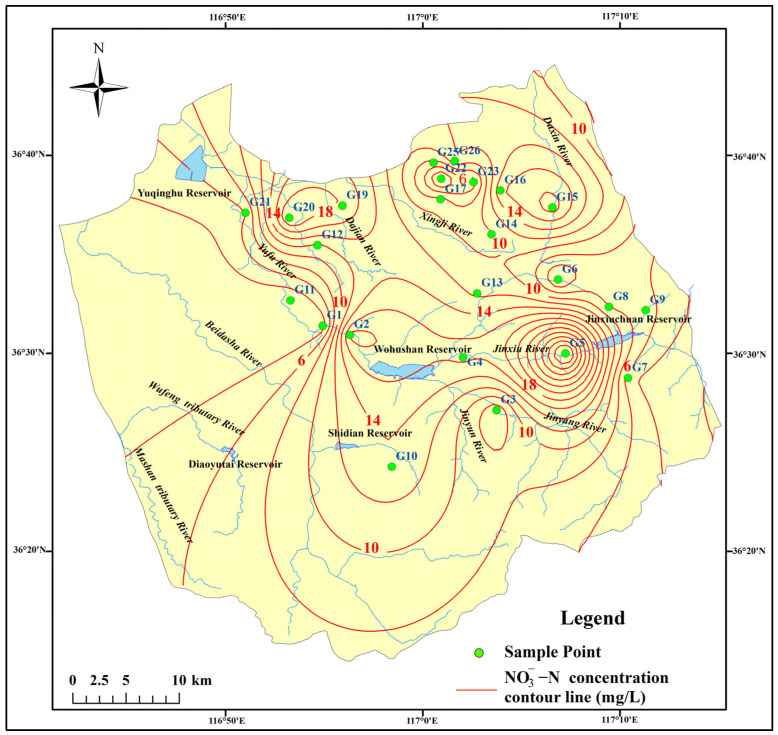
Spatial distribution of NO_3_^−^-N concentrations in groundwater samples.

The spatial distribution of groundwater NO_3_^−^ concentration in the study area was interpolated and analyzed using the Inverse Distance Weighting (IDW) method in ArcGIS 10.3 software. NO_3_^−^ concentration of groundwater exhibits relatively high levels in the sampling points situated in both recharge area and discharge area, while demonstrating lower concentrations in the runoff area (Figure 3). Notably, elevated NO_3_^−^ concentrations were observed at sampling points G2, G4, G5, G15, G16, G19, and G20, with all values exceeding 15 mg/L. Among these, the NO_3_^−^-N concentration at sampling point G5 reached a significant level of 34.47 mg/L. Upon analyzing the sampling point characteristics and surrounding environment, it was determined that G5 is located within a village, with a groundwater depth of approximately 7 m. The well at this site is privately owned and lacks proper wellhead protection infrastructure, allowing domestic sewage and animal waste to infiltrate the aquifer during precipitation events, leading to significant contamination. Other sampling points exhibiting elevated NO_3_^−^ concentrations are similarly located in rural or suburban residential zones, adjacent to agricultural lands and industrial facilities. The elevated NO_3_^−^ levels at these locations may be attributed to industrial pollution and the extensive use of chemical fertilizers.

Spatially, the concentration of NO_3_^−^ in groundwater demonstrates a distinct low–high–low–high variation pattern from the upstream (southern region) to the downstream (northern region) of the study area. Notably, the water samples with NO_3_^−^ concentrations exceeding the standard have formed three distinct high-concentration zones, primarily distributed in both recharge area and discharge area (Table 2).

Land-use types significantly influence the concentration of NO_3_^−^ in both groundwater and surface water [39,40]. The study area features a complex and diverse array of land-use types. The recharge and runoff zones are primarily dedicated to agricultural production, with cultivated land and forested areas comprising over 80% of the total regional area. In contrast, the discharge zone is urban, predominantly consisting of construction land. The southern recharge zone and the central runoff zone are situated in the foothills of Mount Tai, characterized by extensive mountainous terrain with relatively sparse villages and population. Here, the land-use types are mainly secondary forests, shrublands, and cultivated land [41]. Progressing northward along the groundwater flow path towards the suburban areas, the number of villages and population gradually increases, making these areas suitable for habitation and agricultural activities. The land types in these regions are diverse, primarily consisting of farmland, villages, and secondary forests. Analysis of the land-use types at each water sample reveals that 22 out of 30 water samples are located near farmland or villages, accounting for 73.3%. Farmland and villages are areas of intensive human agricultural activities, and the wastewater generated from these areas contains high concentrations of NO_3_^−^. In karst regions, due to the thin soil layer, NO_3_^−^ is easily transported into the soil by rainwater during precipitation, infiltrating groundwater through karst fissures and conduits, and also entering rivers through surface runoff and erosion.

### 3.2. Genesis and Evolution of Hydrochemistry Characteristics

**Figure 4:**
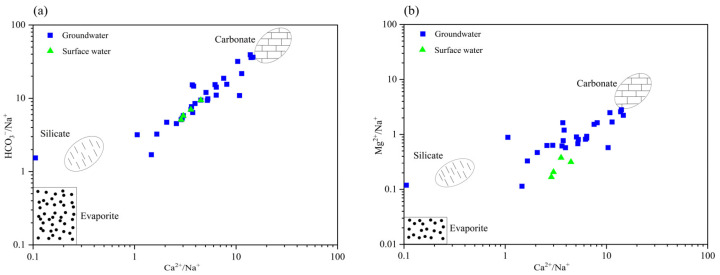
Relationships of HCO_3_/Na and Mg/Na with Ca/Na in groundwater and surface water samples. (**a**) HCO_3_/Na with Ca/Na. (**b**) Mg/Na with Ca/Na.

Through the analysis of HCO_3_/Na vs. Ca/Na and Mg/Na vs. Ca/Na ratios in both surface water and groundwater within the study area, combined with the characteristic ranges of three end-members (carbonate rock, silicate rock, and evaporite) [42], the impact of water–rock interactions on water chemistry was determined. As illustrated in Figure 4, both surface water and groundwater samples cluster near the carbonate rock end-member, with groundwater samples showing a distinct shift towards the upper right quadrant compared to surface water. This spatial distribution pattern suggests that carbonate rock dissolution predominantly controls the ionic composition of both water types, with groundwater exhibiting a more pronounced influence from this process.

**Figure 5:**
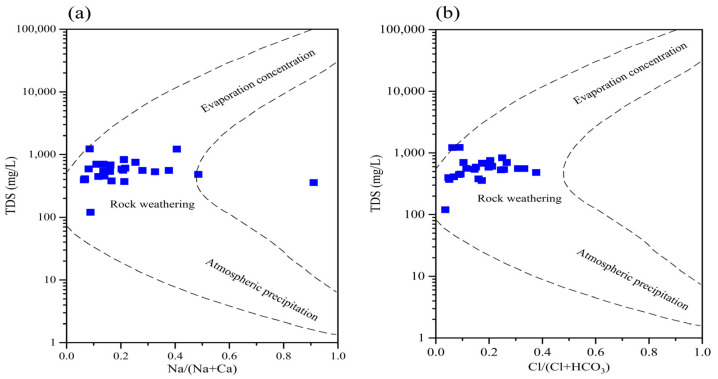
Gibbs diagrams of groundwater sample hydrochemistry. (**a**) TDS with Na/(Na+Ca). (**b**) TDS with Cl/(Cl+HCO_3_).

Gibbs diagrams serve as a crucial analytical tool for characterizing the chemical composition and variation patterns of major ions in groundwater, enabling the identification of dominant processes such as evaporation concentration, rock weathering, and precipitation in the evolution of water chemistry [43]. This methodological approach facilitates a comprehensive understanding of the origin and evolutionary mechanisms of groundwater chemical components in the study area [44,45]. Analysis of the hydrochemical Gibbs diagrams reveals that the majority of groundwater samples are clustered in the central region of the model, exhibiting TDS concentrations ranging from 100 to 1000 mg/L. The ionic ratios, specifically Na/(Na+Ca) and Cl/(Cl+HCO_3_), predominantly fall within the 0 to 0.5 range (Figure 5), suggesting that HCO_3_^−^ and Ca^2+^ constitute the principal anions and cations, respectively. These hydrochemical characteristics are primarily governed by the natural process of rock weathering.

Correlation analysis serves as a valuable tool for evaluating the similarity among groundwater chemical components, thereby elucidating whether different ions originate from common sources [46]. The Pearson linear correlation coefficients for the chemical components (pH, EC, TDS, K^+^, Na^+^, Ca^2^^+^, Mg^2^^+^, Cl^−^, HCO_3_^−^, SO_4_^2^^−^, NO_3_^−^) of groundwater in the study area were calculated using MATLAB 9.3 statistical software. The correlation coefficients, denoted by r, range from −1 to 1. The correlation matrix of groundwater chemical components (Table 3) reveals that TDS demonstrates significant linear correlations with Ca^2^^+^, Mg^2^^+^, Cl^−^, SO_4_^2^^−^, and NO_3_^−^. Notably, the strongest correlations are observed between TDS and Ca^2^^+^ (r = 0.9041) and SO_4_^2^^−^ (r = 0.9130), indicating these components as primary contributors to TDS. While NO_3_^−^ in groundwater typically derives from atmospheric precipitation and soil leaching, anthropogenic activities such as animal waste disposal, domestic sewage discharge, and agricultural practices involving fertilizers and pesticides have substantially elevated NO_3_^−^ concentrations. Both hydrochemical statistics (Table 1) and the correlation matrix (Table 3) confirm that NO_3_^−^ and SO_4_^2^^−^ have emerged as dominant ionic species in the groundwater system. However, the water sample G13 exhibits an abnormally high value of the Na/Na+Ca ratio. This is because the water sample is located beside the river and the sampling depth is relatively shallow. Affected by the mixture of water sources such as surface water, the concentrations of both Ca^2+^ and Mg^2+^ are relatively low.

### 3.3. Nitrate Traceability Analysis

#### 3.3.1. Qualitative Identification of Nitrate Sources

**Figure 6:**
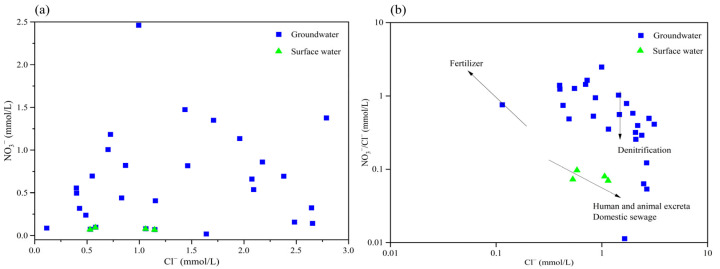
Relationships NO_3_-Cl and NO_3_/Cl-Cl in groundwater and surface water samples. (**a**) NO_3_ with Cl. (**b**) NO_3_/Cl with Cl.

Under normal conditions, the low concentration of NO_3_^−^ in natural groundwater does not pose a significant risk to human health. However, urbanization and agricultural activities, including the extensive use of nitrogen fertilizers, as well as the discharge of human and animal waste and domestic sewage, have resulted in substantial NO_3_^−^ infiltration into aquifers. This phenomenon is particularly pronounced in karst aquifers characterized by a dual structure of surface and subsurface, where infiltration capacity is notably enhanced [47]. Prolonged human exposure to environments with elevated NO_3_^−^ levels can lead to methemoglobinemia and increase the risk of digestive system cancers [19]. Analysis of water chemical components indicates that the NO_3_^−^ concentration in the groundwater of the study area is generally elevated, with some samples surpassing the drinking water safety standards. Given that Cl^−^ in groundwater exhibits greater stability compared to other ionic components, and considering the distinct characteristics of NO_3_^−^ and Cl− from various sources, the NO_3_/Cl ratio serves as a valuable indicator for qualitatively determining the origin of NO_3_^−^. Based on the NO_3_/Cl ratio analysis (Figure 6), it is evident that most water samples in the study area exhibit a NO_3_/Cl ratio below 2, with a wide distribution range of Cl^−^, indicating a low NO_3_/Cl ratio and the variable nature of Cl^−^. Consequently, it can be preliminarily inferred that the nitrate in the study area is mainly affected by human activities.

The method of combining Cl^−^ concentration with the NO_3_/Cl ratio represents one of the most widely employed techniques in hydrochemical studies for identifying NO_3_^−^ sources [48]. Specifically, low Cl^−^ concentrations coupled with high NO_3_/Cl ratios typically indicate that NO_3_^−^ predominantly derives from chemical fertilizers. Conversely, elevated Cl^−^ concentrations accompanied by low NO_3_/Cl ratios generally suggest that NO_3_^−^ primarily originates from human and animal waste, as well as domestic sewage. As illustrated in Figure 6, the relationship between NO_3_^−^ and Cl^−^ demonstrates a weak linear correlation, suggesting that their respective sources are not entirely identical. The water samples are mainly distributed in the regions with low Cl^−^ and high NO_3_/Cl ratios, and the distribution is relatively scattered, suggesting that the NO_3_^−^ in the groundwater and surface water of the study area primarily originates from feces and sewage, and the extent of influence from fertilizers, sewage, and organic manure varies significantly across different regions. The similar distribution of water samples for groundwater and surface water indicates that the two types of water bodies have similar sources of NO_3_^−^.

**Figure 7:**
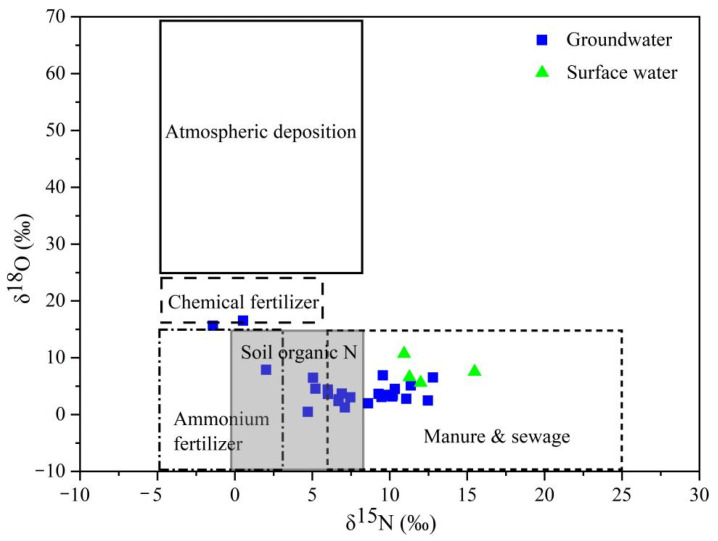
Eigenvalue distribution of nitrate nitrogen and oxygen isotopes.

Under normal conditions, when there is no substantial accumulation of NH_4_^+^ in the environment, the δ^15^N value of NO_3_^−^ produced through mineralization and nitrification aligns with the δ^15^N value of the initial reactants, exhibiting minimal isotopic fractionation [6]. However, significant accumulation of NH_4_^+^ in the environment can lead to incomplete reactions. This can, on one hand, poison the microorganisms, thereby halting or decelerating biochemical reactions; on the other hand, it can cause kinetic isotopic fractionation, resulting in the enrichment of ^14^N in the product NO_3_^−^. In this study, the concentration of NH_4_^+^ in the majority of groundwater samples was found to be extremely low, suggesting that the isotopic fractionation from these reactions is negligible. Given that rainwater contains low levels of NH_4_^+^ and is the primary form of nitrogen, with NO_3_^−^ content also being minimal, rainwater, while diluting groundwater, cannot be the main source of NO_3_^−^ in groundwater. The study area encompasses a vast region, characterized by distinct land-use patterns: the recharge and runoff zones are predominantly utilized for agricultural activities, livestock farming, and artisanal production, whereas the discharge zone is located within the urban center. The intensive application of agricultural fertilizers, combined with the discharge of industrial effluents and domestic wastewater, has resulted in significant point-source contamination. This anthropogenic influence constitutes the principal factor contributing to the marked spatial heterogeneity in NO_3_^−^ concentration distribution. From the characteristic values of nitrate nitrogen and oxygen isotopes in groundwater and surface water in the study area (Figure 7) [49], it is evident that the δ^15^N values range from −1.42‰ to 12.79‰, and the δ^18^O values range from 0.50‰ to 15.63‰. The majority of these values align with the concentration ranges observed in domestic sewage and manure, while a minor proportion corresponds to the ranges characteristic of soil organic nitrogen, atmospheric precipitation, and ammonium-based fertilizers. This distribution pattern strongly suggests that domestic sewage and manure constitute the predominant sources of NO_3_^−^ contamination in both groundwater and surface water within the study area, with soil nitrogen representing a secondary contributor. These findings demonstrate remarkable consistency with the outcomes derived from hydrochemical analyses.

#### 3.3.2. Identification of Denitrification

**Figure 8:**
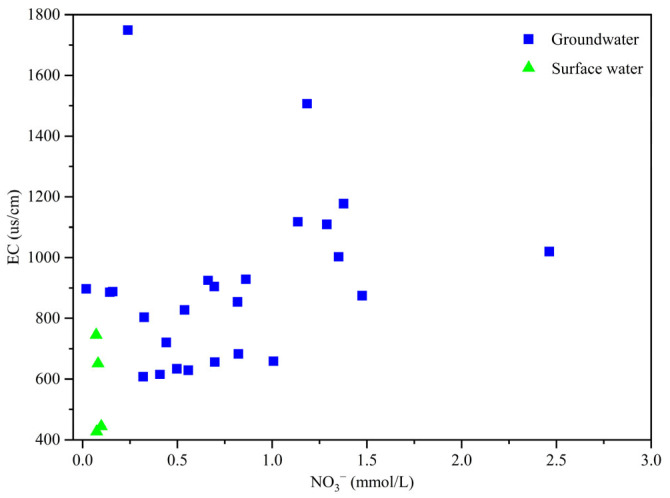
Relationships between EC and NO_3_^−^ in groundwater and surface waters sampled in the study area.

The relationships between Cl^−^ and NO_3_^−^, as well as between EC and NO_3_^−^, can be utilized to preliminarily assess whether variations in NO_3_^−^ concentration in water result from mixing processes without nitrogen form transformation or are induced by denitrification [32]. When an increase in NO_3_^−^ concentration is accompanied by corresponding increases in both Cl^−^ concentration and EC, this suggests that the NO_3_^−^ concentration in water is primarily influenced by mixing processes without nitrogen form transformation. Conversely, the occurrence of denitrification would be indicated by different patterns. As illustrated in Figure 6 and Figure 8, a general trend of increasing Cl^−^ and EC levels with rising NO_3_^−^ concentrations is observed, suggesting that the NO_3_^−^ in the study area is predominantly influenced by mixing processes without nitrogen form transformation.

**Figure 9:**
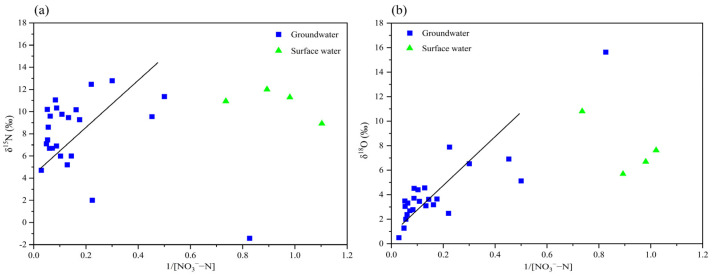
Relationships of δ^15^N and δ^18^O with 1/[NO_3_^−^-N] in groundwater and surface water samples. (**a**) δ^15^N with 1/[NO_3_^−^-N]. (**b**) δ^18^O with 1/[NO_3_^−^-N].

As illustrated in Figure 9, the δ^15^N and δ^18^O values of NO_3_^−^ in both groundwater and surface water within the study area exhibit a positive correlation with the reciprocal of NO_3_^−^-N concentration, suggesting a mixed origin of NO_3_^−^-N. Specifically, the average δ^15^N value for 26 groundwater samples was measured at 7.88‰, while the average δ^15^N value for 4 surface water samples was significantly higher, at 12.40‰. These elevated δ^15^N values align with the characteristic range for fecal matter and sewage, thereby reinforcing the conclusion that these are the predominant sources of NO_3_^−^-N contamination in both groundwater and surface water of the region. The contribution of nitrogen from alternative sources, such as soil nitrogen and fertilizers, appears to be relatively minor, with only a slight possibility of mixing.

The concentration of DO in water serves as a critical parameter for evaluating the self-purification capacity of aquatic systems and represents an essential prerequisite for denitrification processes. Previous studies have demonstrated that the threshold DO concentration for denitrification in groundwater systems is 2 mg/L, with the δ^15^N to δ^18^O ratio associated with denitrification approximating 2:1 [50,51]. In the present investigation, the measured DO concentrations in groundwater samples ranged from 2.32 to 8.69 mg/L (mean = 6.50 mg/L), while surface water samples exhibited DO concentrations between 11.82 and 17.25 mg/L (mean = 13.66 mg/L); δ^15^N in groundwater samples ranged from −1.42‰ to 12.79‰ (mean = 7.88‰), while in surface water samples it ranged from 10.91‰ to 15.47‰ (mean = 12.40‰); δ^18^O in groundwater samples ranged from 0.50‰ to 15.63‰ (mean = 4.27‰), while in surface water samples it ranged from 5.59‰ to 10.80‰ (mean = 7.70‰). These findings suggest that the probability of denitrification occurring in both groundwater and surface water environments within the study area is minimal.

Nitrification is a biochemical process wherein NH_4_^+^ is oxidized to NO_3_^−^ by nitrifying bacteria under aerobic conditions. As an oxygen-dependent reaction, this process involves substantial changes in oxygen isotopes [52]. Consequently, the δ^18^O value of nitrate (δ^18^O_NO3_) serves as a reliable indicator for identifying nitrification processes [53]. In the study area’s groundwater, the δ^18^O_NO3_ values range from 0.50‰ to 15.63‰, with a mean value of 4.27‰. Notably, the majority of these values fall within the −10‰ to 10‰ range, strongly suggesting that nitrification constitutes the predominant mechanism for NO_3_^−^-N transformation in the study area’s groundwater.

#### 3.3.3. Quantitative Analysis of Nitrate Sources

By integrating the δ^15^N_NO3_ and δ^18^O_NO3_ values from both groundwater and surface water in the JSC, the MixSIAR model was utilized to quantify the contribution rates of five potential NO_3_^−^ sources: domestic sewage and manure, soil organic nitrogen, rainfall and fertilizer NH_4_^+^, chemical fertilizer, and atmospheric deposition. The contributions to groundwater were determined to be 37.1%, 25.2%, 21.0%, 11.4%, and 5.3%, respectively, whereas for surface water, the contributions were 56.9%, 14.3%, 10.7%, 10.7%, and 7.4% (Table 4).

The MixSIAR model results demonstrate that domestic sewage and manure constitute the predominant sources of NO_3_^−^ in both groundwater and surface water, with substantial contribution rates of 37.1% and 56.9%, respectively. Secondary sources include soil organic nitrogen, rainfall and fertilizer NH_4_^+^. Furthermore, fertilizer inputs associated with human activities contribute 11.4% and 10.7% to the total NO_3_^−^ sources in groundwater and surface water, respectively, representing relatively minor proportions. Atmospheric deposition exhibits the lowest contribution rate, confirming that domestic sewage and manure are the principal contributors to NO_3_^−^ pollution in the JSC. Collectively, anthropogenic sources of NO_3_^−^ significantly exceed natural sources, highlighting a substantial risk of nitrate pollution in the study area, consistent with previous research findings [54,55].

## 4. Conclusions

The integrated analysis of hydrochemistry and nitrogen–oxygen isotopes, combined with the application of the MixSIAR model, elucidates the spatial variations of nitrate sources and transformations in the Jinan spring catchment. The predominant hydrochemical type was HCO_3_·SO_4_-Ca, with multiple coexisting types indicating a complex hydrochemical system. Significant changes in groundwater hydrochemical conditions in the JSC have been observed, driven by accelerated urbanization and shifts in land-use patterns.

Under the combined influence of natural and anthropogenic factors, the hydrochemical characteristics of both groundwater and surface water in the JSC region have undergone substantial alterations. Groundwater exhibits severe contamination with NO_3_^−^, while surface water maintains lower NO_3_^−^ concentrations and remains free from NO_3_^−^ pollution. Elevated Cl^−^ concentrations and increased EC values suggest the occurrence of point-source pollution in specific localized areas. In both groundwater and surface water systems, NO_3_^−^ primarily originates from domestic sewage and manure, with significant contribution rates of 37.1% and 56.9%, respectively. Soil organic nitrogen serves as a secondary source. The fluctuation in NO_3_^−^ concentration is primarily governed by mixing processes. Within the study area’s groundwater system, nitrification emerges as the dominant process for NO_3_^−^-N transformation, with no substantial evidence of denitrification observed.

The runoff discharge area of the JSC is currently undergoing rapid urbanization, with human activities exerting an increasingly significant impact on groundwater quality. Consequently, in rural residential areas, it is crucial to implement enhanced management of livestock manure, reinforce the construction of sewage pipelines, improve sewage treatment efficiency, and prevent sewage leakage. In agricultural zones, the application of chemical fertilizers should be regulated, and the utilization efficiency of nitrogen fertilizers should be optimized. In urban regions, the treatment and discharge management of domestic sewage must be intensified to mitigate the exacerbation of NO_3_^−^ pollution in the area’s groundwater.

## Figures and Tables

**Table 1:** Hydrochemical characteristics of groundwater and surface waters sampled in the study area.

	Water Samples	*T* /(°C)	pH	EC (µS/cm)	DO	K^+^	Na^+^	Ca^2+^	Mg^2+^	NH_4_^+^−N	Cl^−^	HCO_3_^−^	SO_4_^2−^	NO_2_^−^−N	NO_3_^−^−N	Total Hardness	TDS
					*ρ*/(mg/L)												
Indirect recharge area	G1	24.4	7.57	886	4.75	3.19	42.04	108.41	26.50	0.03	94.24	190.06	173.51	<0.005	2.00	379.87	559.51
	G2	17.9	7.29	1003	6.81	8.28	30.74	160.26	20.92	0.06	60.64	288.79	157.71	<0.005	18.90	486.36	681.89
	G3	24.8	7.2	721	5.74	1.67	18.73	116.17	15.36	0.05	29.47	288.79	87.62	<0.005	6.17	353.35	454.59
	G4	22.7	7.32	1507	6.52	5.27	24.17	261.26	60.28	0.05	25.69	264.11	629.37	0.01	16.57	900.66	1231.09
	G5	17.5	7.25	1020	6.66	2.85	19.67	159.15	32.18	0.02	35.27	306.07	127.82	0.02	34.47	529.95	701.22
	G6	23.9	7.67	1749	7.24	1.82	155.22	227.01	17.78	0.04	17.33	264.11	650.49	0.01	3.33	640.13	1226.05
	G7	23.0	7.9	608	7.48	1.41	18.90	69.95	30.92	<0.01	15.20	288.79	53.42	<0.005	4.46	302.01	372.18
	G8	21.9	7.68	629	8.35	1.03	7.26	106.49	16.21	0.05	14.12	264.11	73.31	<0.005	7.80	332.71	395.28
	G9	25	7.48	634	7.48	0.77	7.34	101.21	19.03	0.06	14.19	288.79	70.68	<0.005	6.95	331.12	399.59
	G10	16.3	7.51	659	8.69	1.59	13.93	105.12	21.32	0.06	24.85	261.64	73.19	<0.005	14.09	350.31	446.96
Direct recharge area	G11	23.3	7.57	888	4.52	2.47	60.69	100.38	19.98	0.03	88.12	197.46	170.47	<0.005	2.21	332.95	560.64
	G12	24.7	7.43	854	7.69	0.45	21.56	137.52	19.97	0.05	51.88	306.07	90.87	<0.005	11.44	425.67	543.68
	G13	21.6	7.6	683	8.07	0.61	96.21	9.46	11.49	0.06	30.79	148.10	71.34	<0.005	11.50	70.94	357.02
	G14	24.6	7.71	905	8.25	1.07	25.05	131.81	20.37	0.05	84.48	246.83	100.75	<0.005	9.72	413.05	540.99
	G15	18.3	7.26	1178	7.48	2.34	46.67	174.43	36.07	0.07	98.96	298.66	224.58	<0.005	19.27	584.12	833.23
	G16	18.7	7.4	1118	7.04	1.63	50.54	148.74	32.13	0.04	69.53	271.51	226.50	<0.005	15.89	503.76	752.80
	G17	21.5	7.24	925	6.15	1.18	35.15	138.21	20.24	0.04	73.67	298.66	112.17	<0.005	9.26	428.50	589.82
	G18	22.8	7.73	656	8.60	0.62	7.47	104.81	21.06	0.04	19.50	269.05	68.87	<0.005	9.75	348.48	411.46
Discharge area	G19	18.3	7.14	1110	5.77	0.38	27.70	176.52	23.79	0.04	110.86	306.07	109.86	<0.005	18.03	538.80	700.83
	G20	20.8	7.32	875	8.02	0.72	13.26	150.47	22.36	0.08	50.96	288.36	97.62	<0.005	20.64	486.82	592.64
	G21	21.6	7.74	615	2.58	1.01	17.85	90.11	16.11	0.05	40.92	214.74	68.49	<0.005	5.70	291.35	380.70
	G22	19.9	6.85	897	2.32	18.13	30.62	116.75	36.82	1.12	58.20	449.23	63.12	<0.005	0.26	443.18	564.68
	G23	22.8	8.03	804	4.92	4.19	48.82	51.79	43.32	0.09	93.89	155.50	138.87	<0.005	4.54	307.73	484.20
	G24	18.1	7.95	204	5.86	1.75	3.48	36.10	2.01	0.05	4.05	111.07	8.43	<0.005	1.21	98.43	119.97
	G25	21.9	7.45	828	6.22	2.73	48.68	100.80	22.86	0.06	74.26	229.55	123.78	<0.005	7.52	345.88	532.96
	G26	20.2	7.35	929	5.70	1.23	37.54	136.51	23.25	0.04	77.24	288.79	121.21	<0.005	12.05	436.63	610.12
Surface water	S1	27.6	8.72	698	17.25	4.86	29.76	85.56	24.62	0.06	40.75	152.54	190.24	<0.005	0.98	286.42	405.26
	S2	30.9	8.56	617	12.98	3.83	23.36	70.54	22.64	0.39	37.69	135.76	151.34	0.09	1.12	269.37	392.35
	S3	24.5	8.62	425	12.58	2.25	11.65	52.35	15.46	0.04	18.86	110.25	88.76	0.01	1.02	208.75	250.26
	S4	26.2	8.67	440	11.82	1.32	12.97	46.53	19.68	0.11	20.66	91.33	102.12	0.02	1.36	197.24	264.03

**Table 2:** Water samples with groundwater concentration of NO_3_^−^-N > 10 mg/L.

Water Samples	NO_3_^−^−N/(mg/L)	Locations
G2	18.90	Indirect recharge area
G4	16.57	Indirect recharge area
G5	34.47	Indirect recharge area
G10	14.09	Indirect recharge area
G12	11.44	Direct recharge area
G13	11.50	Direct recharge area
G15	19.27	Direct recharge area
G16	15.89	Direct recharge area
G19	18.03	Discharge area
G20	20.64	Discharge area
G26	12.05	Discharge area

**Table 3:** Correlation coefficient matrix of parameters and major ions in groundwater samples.

	pH	EC	TDS	K^+^	Na^+^	Ca^2+^	Mg^2+^	HCO_3_^−^	Cl^−^	NO_3_^−^	SO_4_^2−^
pH	1	−0.3915	−0.3823	−0.4588	0.0681	−0.5124	−0.2533	−0.7598	−0.2821	−0.4145	−0.0757
EC	−0.3915	1	0.9825	0.1585	0.6002	0.8625	0.5348	0.3346	0.2852	0.3159	0.8704
TDS	−0.3823	0.9825	1	0.1651	0.5103	0.9041	0.5908	0.3221	0.1913	0.3443	0.9130
K^+^	−0.4588	0.1585	0.1651	1	0.0076	0.1108	0.4103	0.4813	0.1052	−0.1558	0.0756
Na^+^	0.0681	0.6002	0.5103	0.0076	1	0.1719	−0.0187	−0.1796	0.1385	−0.1705	0.5933
Ca^2+^	−0.5124	0.8625	0.9041	0.1108	0.1719	1	0.4885	0.5054	0.1414	0.4514	0.7496
Mg^2+^	−0.2533	0.5348	0.5908	0.4103	−0.0187	0.4885	1	0.3186	0.2684	0.2730	0.4873
HCO_3_^−^	−0.7598	0.3346	0.3221	0.4813	−0.1796	0.5054	0.3186	1	0.0515	0.2671	0.0296
Cl^−^	−0.2821	0.2852	0.1913	0.1052	0.1385	0.1414	0.2684	0.0515	1	0.0993	−0.0415
NO_3_^−^	−0.4145	0.3159	0.3443	−0.1558	−0.1705	0.4514	0.2730	0.2671	0.0993	1	0.0843
SO_4_^2−^	−0.0757	0.8704	0.9130	0.0756	0.5933	0.7496	0.4873	0.0296	−0.0415	0.0843	1

**Table 4:** Contribution rate of potential source to NO_3_^−^ in groundwater and surface water.

Sources	Contribution Rate/%					
	Groundwater			Surface Water		
	Min	Max	Mean ± SD	Min	Max	Mean ± SD
Domestic sewage & manure	8.5	59.4	37.1 ± 13.4	47.7	74.7	56.9 ± 11.0
Soil organic nitrogen	14.0	33.8	25.2 ± 5.3	8.2	17.4	14.3 ± 4.2
NH_4_^+^ in rainfall and fertilizer	10.5	33.5	21.0 ± 5.8	6.1	13.1	10.7 ± 3.2
Chemical fertilizer	5.7	29.5	11.4 ± 5.5	6.6	14.2	10.7 ± 3.1
Atmospheric deposition	1.8	18.5	5.3 ± 3.8	5.7	11.0	7.4 ± 2.4

## Data Availability

The data supporting the findings of this study are available from the corresponding author upon reasonable request.
